# Noncoding RNAs in multiple sclerosis

**DOI:** 10.1186/s13148-018-0586-9

**Published:** 2018-11-29

**Authors:** Xuan Yang, Yuzhang Wu, Bei Zhang, Bing Ni

**Affiliations:** 1 Department of Immunology, Medical College of Qingdao University, 308 Ningxia Road, Shinan District, Qingdao, 266003 China; 2 Department of Pathophysiology, Third Military Medical University, 30 Gaotanyan St., Shapingba District, Chongqing, 400038 China; 3 Institute of Immunology of PLA, Third Military Medical University, 30 Gaotanyan St., Shapingba District, Chongqing, 400038 China

**Keywords:** ncRNAs, lncRNAs, miRNAs, circRNAs, Multiple sclerosis

## Abstract

Multiple sclerosis (MS), a chronic inflammatory demyelinating disease of the central nervous system, is characterized by axonal degeneration and gliosis. Although the causes of MS remain unknown, gene dysregulation in the central nervous system has been associated with the disease pathogenesis. As such, the various regulators of gene expression may be contributing factors. The noncoding (nc) RNAs have piqued the interest of MS researchers due to their known functions in human physiology and various pathological processes, despite being generally characterized as transcripts without apparent protein-coding capacity. Accumulating evidence has indicated that ncRNAs participate in the regulation of MS by acting as epigenetic factors, especially the long (l) ncRNAs and the micro (mi) RNAs, and they are now recognized as key regulatory molecules in MS. In this review, we summarize the most current studies on the contribution of ncRNAs in MS pathogenic processes and discuss their potential applications in the diagnosis and treatment of MS.

## Background

Next-generation sequencing of the human genome revealed the unexpected finding that < 2% of the total genome sequence encodes proteins [1], yet up to 90% of eukaryotic genomes are transcribed, generating noncoding (nc) RNAs that lack an open reading frame and have no protein-coding potential [2, 3]. The ncRNAs are classified according to transcript size; those with length of < 200 nucleotides (nt) are considered small or short ncRNAs—represented by the micro (mi) RNAs that are dominant among small RNAs in eukaryotic cells, the small interfering (si) RNAs and the Piwi-interacting (pi) RNAs ncRNAs—and those with length of > 200 nt are considered long (l) ncRNAs, featuring highly diverse structures and functions [4–6]. Upon their discovery, the ncRNAs were largely dismissed as “transcriptional noise,” but studies have since suggested that this proverbial “dark matter” of the genome may play a major biological role in the development and metabolism of cells and in the pathogenesis of many diseases [7, 8].

Multiple sclerosis (MS) is a chronic inflammatory demyelinating disease of the central nervous system (CNS) and characterized by axonal degeneration and gliosis [9]. The disease affects young adults, mostly between 20 and 40 years old, with a predominance for the female sex. Cases present a wide range of symptoms with varying severity [9, 10]. In general, MS starts with a relapsing-remitting course characterized by sensory disturbances, unilateral optic neuritis, and diplopia. While these signs typically stabilize over a period of several days, the persisting signs of CNS dysfunction become dominant, leading to irreversible disability and cognitive deficits [9]. In addition, there are acute forms of MS, such as the Marburg type, the Balo^,^type (concentric sclerosis), and the Schilder type, all of which are virulent, fulminant and may quickly lead to death [11]. MS can also manifest in childhood, and pediatric MS has a substantial impact on the health-related and overall quality of a lifetime [12].

The causes of MS, while not fully elucidated, involve genetic susceptibility (strongest influence coming from the HLA class II locus) and environmental exposures (such as infectious mononucleosis, Epstein-Barr virus infection and lack of sun exposure/vitamin D) [13]. Although the pathophysiological mechanisms of MS remain largely unknown, studies have implicated autoreactive T cells (primarily, T helper (Th)-1 CD4^+^ T cells and Th17 cells) as being involved, particularly through their secretion of cytokines and activation of the inflammatory cascade; the eventual result is demyelinating plaques—the pathological hallmark of MS. The inflammatory demyelination process triggers microglia activation and chronic oxidative injury, leading to neurodegeneration and, ultimately, axonal and neuronal death [13].

To date, the diagnosis of MS is based upon clinical evidence. In many situations, however, early symptoms of MS can be nonspecific, being suggestive of many other disorders of the CNS. Magnetic resonance imaging can assist in the diagnosis, but a simple specific laboratory test will be much more convenient and affordable for identifying or ruling out MS. A current priority of the MS research field is to find specific biomarkers that will improve the clinical diagnosis of MS and provide further insight into its pathophysiological mechanisms. Considering MS as a typical autoimmune disease and that ncRNAs contribute to immune regulation and pathogenesis of other autoimmune diseases, an increasing number of research groups have sought to identify ncRNAs that can predict the disease activity and its progression.

In this review of the peer-reviewed literature, we present the most recent findings for ncRNAs in MS pathogenesis and discuss the related molecular mechanisms, particularly from the perspective of how these data support the potential of ncRNAs in clinical applications for diagnosis and treatment.

## Biology and primary function of ncRNAs

### MiRNAs

**Fig. 1:**
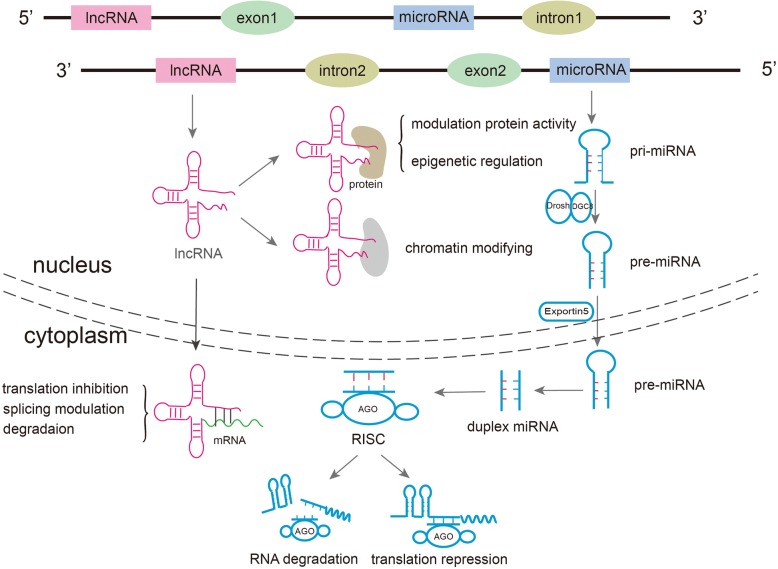
Biogenesis and functions of miRNAs and lncRNAs. MiRNAs are transcribed into pri-miRNAs by RNA Pol II, which are then processed into pre-miRNAs by Drosha in the nucleus. After export to the cytoplasm via exportin 5 and further processing by Dicer into mature double-stranded miRNAs in the cytoplasm, the resultant miRNA duplex is then incorporated into AGO proteins, forming the RISC. In this complex, the enzymatically unwound single-strand miRNAs bind to the 3′-UTRs of target mRNAs, resulting in degradation or translational repression. On the other hand, the biogenesis of lncRNAs can occur in both the nucleus and the cytoplasm, with the transcription usually being mediated by Pol II functioning in various manners, such as chromatin modification and transcriptional and post-transcriptional regulation

MiRNAs are highly expressed in cells of the immune system, CNS and tumors, wherein they regulate the expression of target genes in a sequence-specific manner [14]. The biogenesis of miRNAs begins in the nuclear compartment, where RNA polymerase (Pol) II produces the primary (pri)-miRNA transcripts. The pri-miRNAs are then further processed by the enzyme Drosha and its partner protein DiGeorge syndrome critical region 8 to generate one or more precursor (pre)-miRNAs. The pre-miRNAs are then exported from the nucleus to the cytoplasm by exportin-5/RanGTP, where they are digested by Dicer acting with the trans-activator RNA binding protein. The resultant mature miRNAs (21–25 nt in length; double-stranded duplex) are incorporated into the AGO proteins, forming the RNA-induced silencing complex (RISC). In the RISC, the miRNA duplex is unwound by helicase, and one strand (the “passenger” strand) of this duplex is degraded. The remaining strand (the “guide” strand) then binds to the 3′-untranslated region of its target mRNAs, thereby resulting in degradation or translational repression [15, 16] (Fig. 1).

MiRNA-mediated regulation of gene expression has been implicated in a broad range of biological pathways, such as hematopoiesis, organogenesis, cell differentiation, proliferation, and apoptosis [14, 17–19]. It is estimated that miRNAs target 33% of human genes [20], together forming a complex regulatory network that underlies both physiological and pathological processes, with the potential to both benefit (i.e., normal cell growth) and harm (i.e., cancer) [21]. Indeed, studies have begun to reveal the contributions of miRNAs to the dysregulated gene expressions in human autoimmune diseases, including MS.

### LncRNAs

The lncRNAs have been categorized according to their genomic organization, including in relation to protein coding genes. In addition to the long-standing categories of sense lncRNAs, antisense lncRNAs, intronic lncRNAs, intergenic lncRNAs, and enhancer RNAs [22, 23], the circular (circ) RNAs have been recently identified. This new class of lncRNAs features a covalently closed continuous loop without 5′- or 3′-polarity and has been detected in a wide array of eukaryotic organisms [24–26]. Although the precise origins remain unknown [27], the biogenesis of lncRNAs can occur in either the nuclear or cytoplasmic compartments [28], in contrast to the miRNAs (discussed above). However, like the miRNAs, most lncRNAs are transcribed by Pol II; the others are likely being transcribed by Pol III [23] (Fig. 1).

The collective in-depth studies of lncRNAs have identified an unexpected abundance in the human genome, with several lncRNA databases having been established. The lncRNAs were initially thought to be spurious transcriptional noise resulting from low RNA polymerase fidelity [27, 29], but the advent of high-throughput sequencing has revealed biological functions for many. These include the extensively studied Xist (functioning in X-chromosome inactivation) [30, 31], H19 (genomic imprinting) [32], and HOTAIR (tumor development and progression) [33, 34]. LncRNA-mediated regulation has been identified in almost every step of gene expression; for instance, lncRNAs can regulate gene expression through affecting transcriptional, post-transcriptional, and translational processes; apoptosis; and intracellular trafficking [27, 35–38] by acting as a reservoir of miRNA sponges, protein decoys, and molecular scaffolds [39]. The most recent studies have provided evidence to suggest such functions in autoimmune diseases such as MS [40, 41].

## MiRNAs in MS

### Biomarkers of miRNAs in MS

The development of miRNA profiling techniques has greatly facilitated prospective biomarker studies in MS. To date, a number of miRNA expression profiling studies have been published, in which their potentials as prognostic and diagnostic biomarkers of MS have been evaluated (Table 1).

**Table 1:** MiRNAs dysregulated in MS and possible underlying mechanisms

Source of miRNA	Research model	Change	Target	Function	Ref
PBMC					
hsa-miR-18b, hsa-miR-599, hsa-miR-96	Human	↑	ND	ND	[42]
miR-590	Human	↑	Tob1	Promote Th17 differentiation	[63]
miR-448	Human	↑	PTPN2	Promote Th17 differentiation	[64]
miR-21, miR-146a, miR-146b	Human	↑	ND	ND	[95]
miR-140-5p	Human	↓	STAT1	Inhibit Th1 differentiation	[69]
CD4^+^ T cell					
miR-326	C57BL/6 mice, human	↑	Ets-1	Promote Th17 differentiation	[43]
miR-155	C57BL/6 mice, human	↑	Est-1 and Jarid2	Promote Th17/Th1 differentiation	[59–62]
miRNA let-7e	C57BL/6 mice	↑	IL-10	Promote Th17 differentiation	[65]
miR27a	Human	↑	ND	Inhibit negative regulators of Th17 cell differentiation?	[66]
miR-128, miR-27b, miR-340	C57BL/6 mice human	↑	BMI1? IL-4	Promote Th1 differentiation and inhibit Th2 differentiation	[70]
miR-17-5p	Human	↑	ND	ND	[45]
miR-214	Human	↓	ND	ND	[66]
miR-15b	C57BL/6 mice human	↓	OGT	Inhibit Th17 differentiation	[67]
miR-132	C57BL/6 mice	↓	ND	Suppress T cell proliferation	[68]
B cell					
miR-320a	Human	↓	MMP-9	Disrupt the blood-brain barrier and digest myelin basic protein	[71]
miR-132	Human	↑	Sirtuin-1	ND	[75]
miR-106b-25 cluster, miR-17-92 cluster	Human	↓	PI3K? PTEN?	ND	[74]
Serum/plasma					
miR-326	Human	↑	ND	ND	[51]
miR-614, miR-572, miR-648, miR-1826, miR-422a, miR-22	Human	↑	ND	ND	[52]
miR-24 and miR-137	Human	↑	ND	ND	[47]
miR-15b, miR-23a, miR-223	Human	↓	ND	ND	[48]
miR-155, miR-301a	Human	↓	ND	ND	[51]
miR-1979	Human	↓	ND	ND	[52]
has-miR-let-7a, miR-648a	Human	↓	ND	ND	[50]
Exosome					
miR-15b-5p, miR-23-3p, miR-223-3p, miR-30b-5p, miR-342-3p, miR-432-5p	Human	↑	ND	ND	[53]
Microglia					
miR-155	Human	↑	ND	ND	[77]
miR-124	C57BL/6 mice	↓	ND	Promote the M2 phenotype of macrophages and microglia	[76]
Active lesions					
miR-34a, miR-155, miR-326	Human	↑	CD47	Promote phagocytosis of myelin	[80]
CSF					
miR-922	Human	↓	ND	ND	[54]
miR-181c, miR-633	Human	↑			
miR-219	Human	↓	ND	ND	[55]
miR-150	Human	↑	ND	ND	[56]
Demyelinated MS hippocampi					
miR-124	Human	↑	AMPA2	ND	[96]
			AMPA3		

*ND* not determined; ↑, upregulation; ↓, downregulation;?, presumed

Studies of blood from MS patients have yielded differential miRNA expression profiles with relation to disease status. The first study [42] analyzed the expression patterns of 364 miRNAs in peripheral blood mononuclear cells (PBMCs) from MS patients in relapse and in remission, as well as in healthy controls. A relapse phase-specific miRNA signature was found, showing strong dysregulation of miR-18b and miR-599. The remission phase-specific miRNA signature showed a strong dysregulation of miR-96. The study also yielded a set of miRNAs considered good candidates for future biomarker studies in MS and at least two more miRNAs with good potential for characterizing the relapse status, even though the exact mechanism of the latter remains unclear. Subsequent studies confirmed altered expression of the MS-related miRNAs miR-326, hsa-miR-145, and miR-17-5p in PBMCs and CD4^+^ T cells of relapsing MS patients [43–45]. In addition, 23 MS-related differentially expressed miRNAs were found to be related to a predominance of upregulated genes in CD4^+^ regulatory T cells of patients [46].

MS-related differential expression of miRNAs have also been observed in patient sera. Such a detection method is particularly attractive as a convenient means for diagnosing or prognosing disease cases [47–52]. The miRNAs with promise for such research and development include miR-15b, miR-23a and miR-223 (significantly decreased in MS sera vs healthy controls), miR-155 and miR-301a (decreased), and miR-326 (increased in relapsing-remitting (RR) MS sera) [51]. In addition, some exosomal miRNAs in sera have been identified as differentially expressed between RRMS and progressive MS [53]. Ultimately, comparisons between the two clinically distinct MS subtypes—RRMS and progressive MS—identified nine miRNAs with significant differential expression, namely miR-15b-5p, miR-23a-3p, miR-223-3p, miR-374a-5p, miR-30b-5p, miR-433-3p, miR-485-3p, miR-342-3p, and miR-432-5p. The finding of miRNAs associated with circulating exosomes suggests a potential for their development as informative biomarkers, not only for distinguishing MS cases from healthy controls but also in predicting disease subtype with accuracy.

According to the MS characteristic as a demyelinating neurodegenerative disorder, disease-specific miRNA biomarkers in cerebrospinal fluid (CSF) could also be of great significance. In an analysis of miRNAs in CSF of MS patients, Haghikia et al. [54] found that miR-922, miR-181c, and miR-633 were differentially regulated, as compared to expression levels detected in patients with other neurologic diseases. In a more recent focused investigation of the levels of miR-219 in CSF in relation to MS diagnosis, MS patients were found to have the highest rate of undetectable miR-219 compared to controls; in addition, a strong positive association was found between the undetectable level of miR-219 and diagnosis of MS, suggesting its potential as a biomarker for MS diagnosis [55]. Yet, another study in recent years identified miR-150 in CSF as a putative novel biomarker of active inflammatory disease, suggesting its potential for early diagnosis of MS [56].

Interestingly, infectious pathogens can produce miRNAs in patient serum, easily detectable by standard PCR. For instance, the CNS- and gut-selective immunosuppressant natalizumab is one of the most effective therapies for active RRMS, but its long-term use is associated with development of progressive multifocal leucoencephalopathy, a serious opportunistic brain infection caused by a neurotropic strain of the JC virus [57]. When Basnyat and colleagues [58] investigated plasma of natalizumab-treated MS patients, they determined that the presence of JC polyoma virus miRNA in plasma may indicate asymptomatic viral infection and concluded that the virus-encoded miRNAs may hold promise as risk assessment biomarkers for progressive multifocal leucoencephalopathy in MS.

### MiRNAs involved in MS pathogenesis

#### Regulation of immune cells

**Fig. 2:**
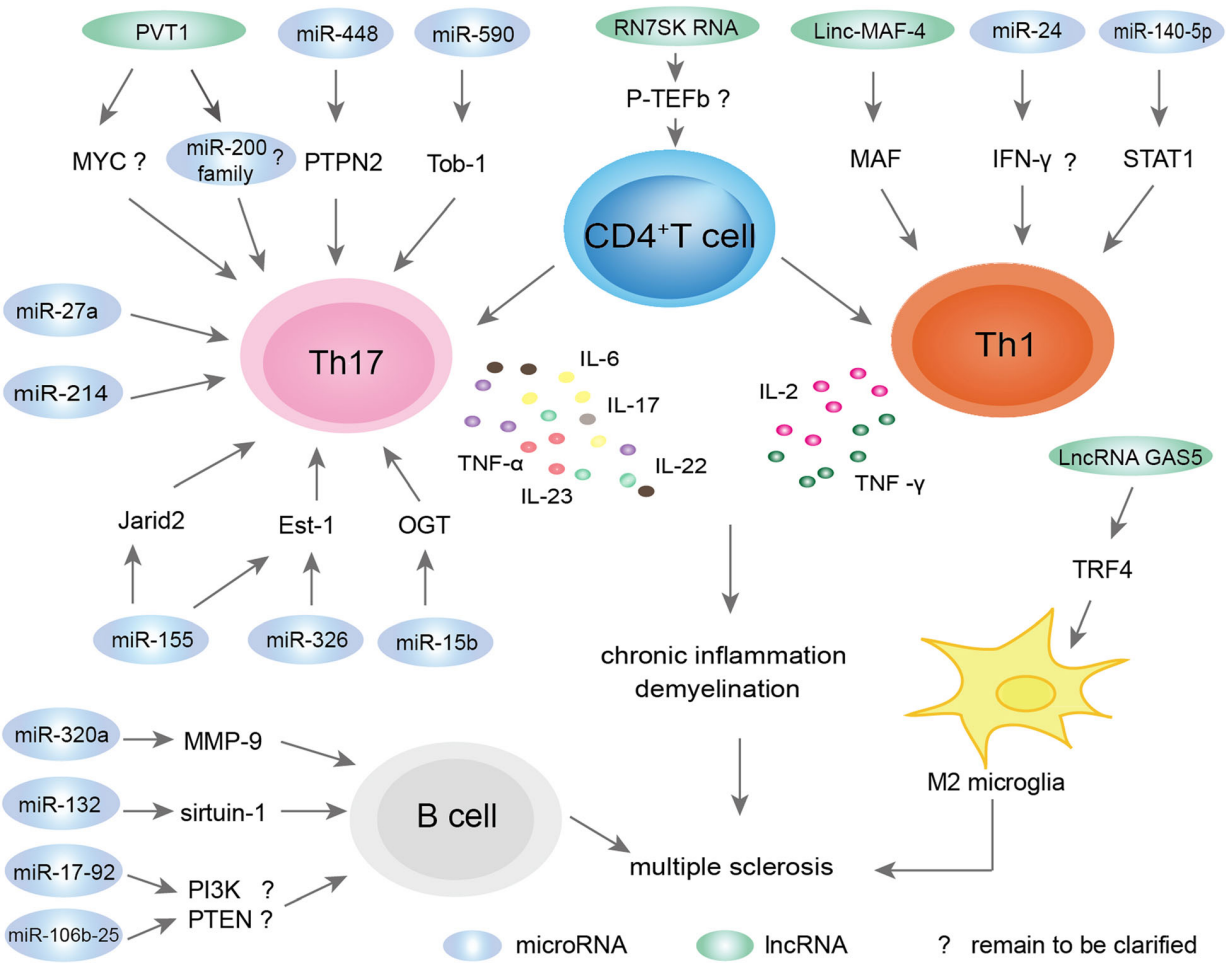
Mechanisms of ncRNAs in MS. One of the major pathophysiological mechanisms of MS involves autoreactive T cells, primarily Th1 and Th17 cells, leading to cytokine secretion and activation of an inflammatory cascade. These changes eventually result in demyelination within the brain and spinal cord, and axonal damage. Emerging lines of evidence have demonstrated that ncRNAs (miRNAs and lncRNAs) are involved in MS pathogenesis, functioning in modulation of CD4^+^ T cell activity, including upregulating activity of the proinflammatory Th1 cells and Th17 cells. The pathophysiological mechanisms of MS that involve B cells are also shown here

Proinflammatory responses mediate autoimmune demyelination in MS. As such, the potential effects and underlying mechanisms of miRNAs have been investigated extensively for the MS-related immune cell types, namely the CD4^+^ T cells, B cells, and macrophages (Fig. 2).

##### Th17 and Th1 cells

Increasing evidence has indicated that miRNA might exert their effects on Th17/Th1 cells by influencing their differentiation and function. The MS-related upregulated miRNAs can inhibit negative regulators of Th17/Th1 cell differentiation, and vice versa for the downregulated miRNAs. In the pioneer study about the role of miRNAs in T cells in MS, Du et al. [43] found that miR-326 expression was associated with IL-17–producing Th17 cells, key factors in the pathogenesis of MS. Specifically, miR-326 was found to be overexpressed in Th17 cells of patients with RRMS and to promote Th17 cell differentiation by inhibiting Ets-1, a negative regulator of Th-17 differentiation. In other studies, increased miR-155 expression was found within T cells and shown to promote Th17/Th1 differentiation similarly, by targeting the transcription factor Ets-1 [59–61]. Finally, the miR-155 was found to control Th17 cell function by suppressing the inhibitory effects of Jarid2, a DNA-binding protein that recruits the chromatin modifier polycomb repressive complex 2 to the chromatin [62].

The increased miRNAs in MS might promote Th17 differentiation through other mechanisms as well. Liu et al. [63] reported that miR-590 was increased in relapse cases of MS and demonstrated that miR-590 promotes pathogenic Th17 cell differentiation through targeting of Tob1, a member of the tob/btg1 family of antiproliferative proteins. They also found that the overexpression of miR-590 increased the pathogenicity of Th17 cells by upregulating several inflammation-associated molecules, such as CXCL3, CSF2, and IL-23R. Wu et al. [64] reported that miR-448 was significantly increased in MS patients and demonstrated its promotion of MS development through induction of the Th17 response by targeting the protein tyrosine phosphatase non-receptor type 2 (PTPN2). Guan et al. [65] reported that the level of miRNA let-7e was significantly upregulated in the experimental autoimmune encephalomyelitis (EAE) model of MS, showing the expression as being mainly in CD4^+^ T cells and with function in enhancement of Th1 and Th17 cells to aggravate EAE, probably by targeting IL-10. Finally, Ahmadian-Elmi et al. [66] demonstrated that miR-27a was increased in relapsing cases of MS and suggested that such may inhibit the negative regulators of Th17 cell differentiation.

Downregulated miRNAs in MS have also been reported and appear to influence Th17/Th1 differentiation similar to the upregulated miRNAs. For example, Liu et al. [67] reported downregulated expression of miR-15b in CD4^+^ T cells obtained from patients with MS and mice with EAE, and demonstrated a consequent inhibition of Th17 differentiation through targeting of O-linked *N*-acetylglucosamine transferase (OGT), with subsequent effects on the transcriptional regulation of RORγt through O-GlcNAcylation of NF-κB. Hanieh et al. [68] reported that the expression of miR-132 was downregulated in CD4^+^ cells and associated with EAE severity; miR-132 silencing in vivo abolished 2,3,7,8-tetrachlorodibenzo-p-dioxin-induced cholinergic anti-inflammation and aggravated EAE, while its overexpression in encephalitogenic CD4^+^ T cells decreased IL-17 and IFN-γ and suppressed T cell proliferation, indicating its effects on the functions of Th1 and Th17 cells. Guan et al. [69] demonstrated that the miR-140-5p was markedly downregulated in MS, and results of further investigation strongly suggested that this miRNA inhibits Th1 differentiation through downregulation of the signal transducer and activator of transcription 1 (STAT1) gene. As such, the downregulation of miR-140-5p leads to enhanced development of Th1 cells and MS disease severity. Finally, the study by Ahmadian-Elmi et al. [66] discussed above also found that miR-214 was downregulated in the relapsing phase of MS and theorized to inhibit Th17 differentiation in MS.

##### Naïve and memory CD4^+^ T cells

In a study of CD4^+^ T cells from MS patients, Guerau-de-Arellano and colleages [70] found increased miR-128 and miR-27b in the naïve cell subset and miR-340 in the memory cell subset. These miRNAs were also found to synergistically promote Th1 differentiation and inhibit Th2 cell differentiation. Moreover, the underlying mechanisms were shown to involve direct suppression of the expression of B lymphoma Mo-MLV insertion region 1 homolog (BMI1) and IL-4, resulting in decreased GATA3 levels and the imbalance of Th2 and Th1 populations. The study applied this knowledge as treatment in vitro, exposing MS patient T cells to inhibitors of these miRNAs, with the result of restoring the Th2 responses.

##### B cells

Similar to the findings in T cells, studies of dysregulated miRNAs in B cells also uncovered contributions to the pathogenesis of MS. Aung et al. [71] reported that miR-320a was significantly downregulated in B cells of MS patients and demonstrated that matrix metallopeptidase-9 (MMP-9) was consequently increased significantly in B cells. MMP-9 is able to disrupt the blood-brain barrier and to digest myelin basic protein [72, 73]; increased expression and secretion of MMP-9 in B cells may contribute to damaging the blood-brain barrier and myelin destruction, thereby contributing to MS pathogenesis. In addition, Sievers et al. [74] found 10 distinct differentially expressed miRNAs in B cells from untreated MS patients compared with natalizumab-treated MS patients; among these miRNAs, the miR-106b-25 cluster and miR-17-92 cluster were particularly deregulated. Furthermore, miRNA-mRNA interaction analysis revealed that B cell receptor, phosphatidyl-inositol-3-kinase (PI3K), and phosphatase and tensin homology (PTEN) are the most affected signaling pathways in B cells of MS patients. Finally, Miyazaki et al. [75] found overexpression of miR-132 in B cells of MS patients and speculated that a novel miRNA-132/surtuin-1 axis may underlie the aberrant B cell cytokine regulation in patients with RRMS.

##### Macrophages

Macrophages and microglial cells (considered as CNS-resident macrophages) may also be involved in MS [76]. The expression of miR-155 was reported as significantly increased in both peripheral circulating CD14^+^ monocytes and CD68^+^ cells of active lesions from MS patients compared to those taken from control donors [77]. Ponomarev et al. [78] reported that miR-124 was downregulated in activated microglia during EAE. MiR-124 is a key regulator of microglia quiescence in the CNS and, thus, is an important modulator of monocyte and macrophage activation, contributing to the M2 phenotype of macrophages and microglia in the periphery during EAE. In the same study, transfection of macrophages with miR-124 was found to cause downregulation of M1-associated effector molecules, such as TNF-α, and upregulation of M2-related effector molecules, such as TGF-β, arginase-1, and FIZZ1 [78]; these changes would confer the M2 phenotype in MS, establishing a profile crucial for the suppression of EAE.

##### PBMCs

Genetic susceptibility is highly correlated with the etiology of MS. Most recently, Luo et al. [79] identified 21 differentially expressed miRNAs in PBMCs from MS patients, as compared with PBMCs from healthy controls; these included miR-199a and miR-142-3p. By using biological information analysis, they constructed a network of these miRNAs and their susceptibility genes and found that KRAS (a vital MS susceptibility gene) is a predictive target of miR-199a. In addition, miR-142-3p (a key negative regulator of IL-1β-dependent synaptopathy in neuroinflammation) was predicted to target both IL7R and KRAS genes. Collectively, these results suggest that miR-199a, miR-142-3p, and their target genes—particularly the IL7R and KRAS genes—might serve as MS therapeutic targets in the MAPK /JAK-STAT signaling transduction pathway.

#### Direct influence of CNS cells

In recent years, multiple studies have identified miRNAs with high-level expression in the CNS under physiologic conditions. Characterization of these miRNAs has revealed functions in establishing an environment that supports remyelination and axon regeneration. Based on the characteristics of MS, understanding the dysregulation and functions of miRNAs in the CNS will likely benefit our understanding of the pathogenesis of MS and translation of that knowledge to clinical applications.

Astrocytes are one of the cell types participating in MS plaque formation. Using laser capture microdissection to analyze cell type-specific miRNA profiles, Junker et al. [80] determined that astrocytes contained all 10 of the miRNAs that were most strongly upregulated in active MS lesions. Among these 10, miR-155, miR-34a, and miR-326 were predicted to target CD47, ultimately serving to release macrophages from inhibitory control and subsequently promoting phagocytosis of myelin [80].

Damage to oligodendrocytes (OLs) can lead to demyelination and hinder effective neural communication, both being features associated with MS. A murine-based study by Dugas et al. [81] indicated that multiple miRNAs might act as a positive feedback loop to coordinate rapid transition of gene expression during OL differentiation (i.e., miR-219, miR-338, miR-23, and miR-9), proliferation (i.e., miR-17-5p and miR-19b), and myelination at multiple stages of the disease process. The study also characterized the role of miR-219 in maintenance of lipids and redox homeostasis in mature OLs, showing the involvement of binding to ELOVL7 and indicating that persistent miRNA expression is required for maintenance of the myelin sheath [81]. The essential roles of miR-17-5p and miR-19b in controlling the number of OLs, through targeting of PTEN (an inhibitor of PI3K signaling, and a negative regulator of cell proliferation in RRMS patients), had been shown by another group [45]. In addition, miR-23 regulation of lamin B1 was demonstrated as crucial for OL development and myelination by an earlier study [82], supporting its potential role in the pathogenic mechanism of MS.

## LncRNAs in MS

### MS-related lncRNAs as biomarkers

The lncRNAs and their abilities to control expression of genes, as well as their contributions to pathogeneses of diseases, have only recently been recognized. Although the research into lncRNAs’ involvement in MS, in particular, is only in its infancy, aberrant lncRNA expression has been observed in human cases.

Santoro et al. [83] identified three lncRNAs upregulated in the serum of RRMS patients, namely the nuclear paraspeckle assembly transcript 1 (NEAT1), taurine upregulated 1 (TUG1), and 7SK small nuclear (RN7SK RNA). Zhang et al. [84] further investigated the expression of lncRNAs in PBMCs of patients with MS and identified six aberrant lncRNAs, consisting of three upregulated (LncRNA ENSG00000231898.3, lncRNA XLOC_009626, and lncRNA XLOC_010881) and three downregulated (LncRNA ENSG00000233392.1, lncRNA ENSG00000259906.1, and lncRNA XLOC_010931). Last year, Eftekharian et al. [85] identified another three aberrantly expressed lncRNAs in the circulating blood cells of RRMS patients, as compared to samples from healthy controls; these included downregulated lncRNAs (PVT1 and FAS-AS1) and an upregulated lncRNA (THRIL). Although it remains unknown how these lncRNAs may be involved in MS pathogenesis, their aberrant expression profile suggests their candidacy as MS-specific biomarkers for predication of disease course or treatment response (Table 2).

**Table 2:** Dysregulated lncRNAs in MS

Source of lncRNA	Research model	Change	Target	Function	Ref
PBMC					
THRIL	Human	↑	ND	ND	[85, 97, 98]
FAS-AS1	Human	↓	ND	ND	
PVT1	Human	↓	MYC, miR-200 family	ND	
lncRNA ENSG00000231898.3	Human	↑	ND	ND	[84]
lncRNA XLOC_009626	Human	↑	ND	ND	[84]
lncRNA XLOC_010881	Human	↑	ND	ND	[84]
lncRNA ENSG00000233392.1	Human	↓	ND	ND	[84]
lncRNA ENSG00000259906.1	Human	↓	ND	ND	[84]
lncRNA XLOC_010931	Human	↓	ND	ND	[84]
linc-MAF-4	Human	↑	MAF	↑ Th1 differentiation, ↓ Th2 differentiation	[91]
Serum					
NEAT1	Human	↑	ND	ND	[83]
TUG1	Human	↑	ND	ND	[83]
RN7SK RNA	Human	↑	PTEF-b?	ND	[83]
M2-type microglia					
lncRNA GAS5	C57BL/6 mice	↑	TRF4	↓ M2 polarization	[87]

*ND* not determined; ↑, upregulation; ↓, down-regulation;?, presumed

### LncRNAs involved in MS pathogenesis

Recently, several lncRNAs have been verified as involved in MS pathogenesis (Table 2 and Fig. 2). It is well known that a proinflammation response is the primary cause of MS development and that a cellular profile of high-M1 versus low-M2 polarized microglia is a pivotal feature of MS pathogenesis [86]. As such, Sun et al. [87] performed a microarray screen and found that the lncRNA GAS5 was significantly upregulated in amoeboid-shaped microglia of MS patients and that this feature was significantly associated with MS. Subsequent functional studies in mice with EAE revealed that lncRNA GAS5 suppresses the transcription of TRF4, a key factor controlling M2 polarization, by recruiting the polycomb repressive complex 2, thereby inhibiting M2 polarization. Furthermore, interference with lncRNA GAS5 in transplanted microglia was found to attenuate the progression of EAE and to promote remyelination, suggesting this lncRNA as a promising target for MS treatment.

Increasing evidence supports the involvement of the proinflammatory cells Th1 and Th17 in the disease course of MS [88]. As such, interfering with the Th cell population, such as by expansion of Th2 cells or inhibition of Th1/Th17 cells, was hypothesized to help resolve MS; indeed, murine model studies showed that these approaches ameliorated EAE [89, 90]. Last year, Zhang et al. [91] reported that linc-MAF-4 was increased significantly in PBMCs of patients with MS, as compared to healthy controls, and that increasing the levels significantly facilitated Th1 differentiation and inhibited Th2 differentiation by directly inhibiting MAF, a Th2 cell transcription factor. In contrast, downregulation of linc-MAF-4 was found to inhibit development of Th1 cells and to heighten the development of Th2 cells. Therefore, by targeting MAF to regulate Th1/Th2 differentiation, linc-MAF-4 increased the in vivo Th1/Th2 ratio and promoted MS pathogenesis, suggesting its potential as a therapeutic target for MS.

## CircRNAs in MS

CircRNAs, a novel family of ncRNAs, have emerged as the newest player in the complex network of gene expression regulation. Though circRNAs have been implicated in several types of diseases [92], there are only two published studies to date involving circRNAs analysis in MS patients. While investigating alternative splicing abnormalities in the GSDMB gene, Cardamone et al. [93] found an upregulated circRNA (hsa_circ_0106803) in the PBMCs of RRMS patients and identified resultant novel isoforms of the GSDMB gene. Almost at the same time, Iparraguirre et al. [94] were carrying out circRNA expression profiling of peripheral blood leucocytes from MS patients and healthy controls; the comparative analysis and subsequent validation experiments revealed that circ_0005402 and circ_0035560 are downregulated in MS patients. While it remains unknown whether these MS-related circRNAs are involved in the process of MS development, the data support their further study as blood biomarkers for MS disease.

## Conclusion and perspectives

The pathophysiological and clinical complexity of MS underlies the urgent need for a great variety of potential biomarkers specific for diagnosis, prediction of disease course, and treatment. Emerging evidence indicates the potential of ncRNAs in the regulation of gene expression, providing new opportunities to understand the course of various diseases, including MS. Indeed, multiple ncRNAs have been found to be expressed differentially in diseased patients compared with healthy controls, and some ncRNAs have been verified as involved in MS pathogenesis, through various mechanisms. The collective findings have served to indicate their potential value as diagnostic and predictive markers in MS (Table 3).

**Table 3:** Published values of Sn, Sp, and AUC for biomarker candidates for MS

NcRNA subtype within	NcRNA name	Sn	Sp	AUC	Ref
MicroRNAs					
Blood cells	hsa-miR-145	90.0%	89.5%	0.96	[44]
Serum/plasma	miR-223	ND	ND	0.80	[48]
	miR-15b	ND	ND	0.75	[48]
	miR-24	ND	ND	0.686	[47]
	miR-137	ND	ND	0.741	[47]
Exosome	miR-15b-5p	ND	ND	0.76	[53]
	miR-23a-3p	ND	ND	0.80	[53]
	miR-223-3p	ND	ND	0.77	[53]
	miR-374a-5p	ND	ND	0.78	[53]
	miR-30b-5p	ND	ND	0.82	[53]
	miR-433-3p	ND	ND	0.93	[53]
	miR-485-5p	ND	ND	0.87	[53]
	miR-342-3p	ND	ND	0.81	[53]
	miR-432-5p	ND	ND	0.86	[53]
CSF	miR-181c	ND	ND	0.73	[54]
	miR-922	ND	ND	0.74	[54]
	miR-633	ND	ND	0.82	[54]
	miR-150	89%	50%	0.744	[56]
CircRNAs					
PBMCs	circ_0005402	94.4%	75.0%	0.899	[94]
	circ_0035560	55%	88.9%	0.706	[94]

*Sn* sensibility, *Sp* specificity, *AUC* area under the curve, *PBMCs* peripheral blood mononuclear cells, *ND* not determined

The field of research exploring ncRNAs in MS, however, requires further attention, particularly for confirmation before the findings can be translated to clinical applications; this is especially the case for the circRNAs, the newest promising ncRNAs subtype, which have not been explored extensively in MS yet which might be one of the key research goals in next few years. In addition, the exact effects of various ncRNAs on the course of diseases need to be clarified. The key underlying mechanisms, including sponging and RNA and protein binding for disease-specific ncRNAs, have to be explored comprehensively. Furthermore, novel mechanisms such as protein translation capacity of circRNAs and other unknown mechanisms remain to be explored, although such knowledge will expand our understanding of the essence of diseases and more importantly facilitate the design of reasonable therapeutic strategies against various diseases that threaten human health.

## Abbreviations

circRNA: Circular RNAs
CNS: Central nervous system
EAE: Experimental autoimmune encephalomyelitis
lncRNAs: Long ncRNAs
miRNAs: Micro RNAs
MMP-9: Matrix metallopeptidase-9
MS: Multiple sclerosis
ncRNAs: Noncoding RNAs
NEAT1: Nuclear paraspeckle assembly transcript 1
nt: Nucleotides
PBMCs: Peripheral blood mononuclear cells
Pol: RNA polymerase
pre-miRNAs: Precursor miRNAs
pri-miRNAs: Primary miRNA
PTPN2: Tyrosine phosphatase non-receptor type 2
RN7SK RNA: 7SK small nuclear
RRMS: Relapsing-remitting MS
Th: T helper
TUG1: Taurine upregulated 1
